# Case report: Coexistence of sigmoid tumor with unusual pathological features and multiple colorectal neuroendocrine tumors with lymph node metastases

**DOI:** 10.3389/fonc.2023.1073234

**Published:** 2023-03-13

**Authors:** Shu Pang, Jiugang Song, Kun Zhang, Jia Wang, Haiying Zhao, Yongjun Wang, Peng Li, Ye Zong, Yongdong Wu

**Affiliations:** ^1^ Department of General Practice, Beijing Friendship Hospital, Capital Medical University, Beijing, China; ^2^ Department of Gastroenterology, Beijing Friendship Hospital, Capital Medical University, Beijing, China; ^3^ Department of Pathology, Beijing Friendship Hospital, Capital Medical University, Beijing, China; ^4^ Department of Ultrasonography, Beijing Friendship Hospital, Capital Medical University, Beijing, China

**Keywords:** sigmoid neoplasm, multiple colorectal neuroendocrine tumors, lymph nodes metastases, neuroendocrine, adenocarcinoma, case report

## Abstract

The coexistence of adenocarcinoma and neuroendocrine neoplasm (NEN) in the same tumor is rare. What is rarer is that the neuroendocrine component is a well-differentiated neuroendocrine tumor (NET) Grade (G) 1. Most colorectal NETs are single, but multiple neuroendocrine tumors (M-NETs) are rare. Well-differentiated NETs rarely metastasize. Here, we present a unique case of a synchronous sigmoid tumor and multiple colorectal NETs with lymph node metastases. The sigmoid tumor consisted of adenocarcinoma and NET G1. The metastatic component was NET G1. A 64-year-old man underwent a colonoscopy for persistent changes in bowel habits and positive fecal occult blood for 1 year. An ulcerative lesion, which was diagnosed as colon cancer, was seen in the sigmoid colon. In addition, scattered lesions could be seen in the colon and rectum. Surgical resection was performed. Pathological findings suggested that the ulcerative lesion was composed of 80% adenocarcinoma and 20% neuroendocrine component (NET G1), while the remaining lesions were consistent with NET G1. At the same time, 11 lymph nodes around the resected intestinal segment were invaded by NET G1. The prognosis of the patient was good. After 13 months of follow-up, no recurrence and no metastasis were found. We hope to provide a reference and improve our understanding of the clinicopathological features and biological behavior of these unique tumors. We also aim to emphasize the importance of radical surgery and individualized treatment.

## 1 Introduction

Colorectal cancer (CRC) is one of the major causes of cancer-related deaths. The most common histologic subtype of CRC is adenocarcinoma (1). According to the 2019 World Health Organization (WHO) classification, the neuroendocrine neoplasm (NEN) is subdivided into well-differentiated neuroendocrine tumor (NET; i.e., NET Grade (G) 1, NET G2, and NET G3) and poorly differentiated neuroendocrine carcinoma (NEC) (2). The coexistence of adenocarcinoma and NEN in one tumor is rare. Even rarer is the presence of a well-differentiated NET G1 component. Most colorectal NETs are single, and multiple neuroendocrine tumors (M-NETs) are rare (3). Lymph node metastasis of NETs is related to the degree of differentiation. Well-differentiated NETs rarely metastasize. This article reports an interesting case of synchronous sigmoid tumor consisting of 80% adenocarcinoma and 20% neuroendocrine component (NET G1) and multiple colorectal NETs with lymph node metastases. The sigmoid tumor presented with diffusely metastatic lymph nodes with NET G1 component, which is unique. Radical surgical resection and individualized treatment usually imply a good prognosis. We hope to provide a reference and improve our understanding of the clinicopathological features and biological behavior of these unique tumors.

## 2 Case presentation

**Figure 1:**
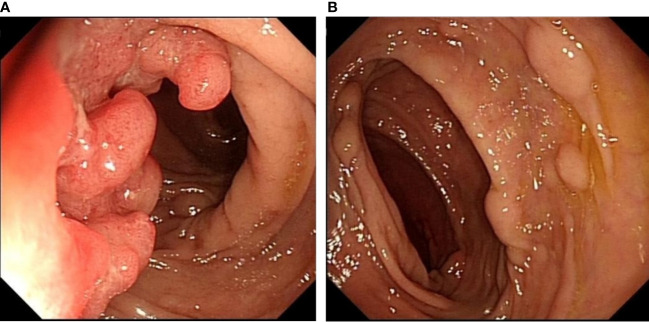
Colonoscopy showing an ulcerative mass, approximately 3 * 3 cm in size, located in the sigmoid colon 15 cm proximal to the anal verge **(A)**. Scattered raised lesions with a size of 0.4–1.0 cm located in the colon and rectum **(B)**.

A 64-year-old man presented to our hospital with persistent changes in bowel habits and positive fecal occult blood for 1 year in July 2021. His weight had dropped by approximately 10 kg since the disease onset. Personal and family history was not contributory. There was no previous history of intestinal inflammatory diseases or other related disease history. No obvious mass was found in the digital rectal examination, and no blood was found on the finger pad. Blood routine examination, biochemical examination, and tumor markers showed no obvious abnormalities. We did not check the levels of urinary 5-hydroxyindoleacetic acid or plasma serotonin because a NET was not suspected at that time. Colonoscopy was performed, and an ulcerative mass, approximately 3 * 3 cm in size, was seen in the sigmoid colon 15 cm proximal to the anal verge (**Figure 1A**), which was diagnosed as colon cancer. In addition, scattered lesions with a size of 0.4–1.0 cm could be seen in the colon and rectum (**Figure 1B**). A biopsy was performed, and histology revealed that the ulcerative mass was an adenocarcinoma and that the remaining lesions were NETs (G1). Abdominal and pelvic enhanced CT showed that the wall of the junction area between the rectum and sigmoid colon was thickened with abnormal enhancement, and multiple small lymph nodes were found in the mesorectum. Malignant tumors with lymph node metastasis might be considered. Based on the above findings, we considered that surgical resection was necessary.

**Figure 2:**
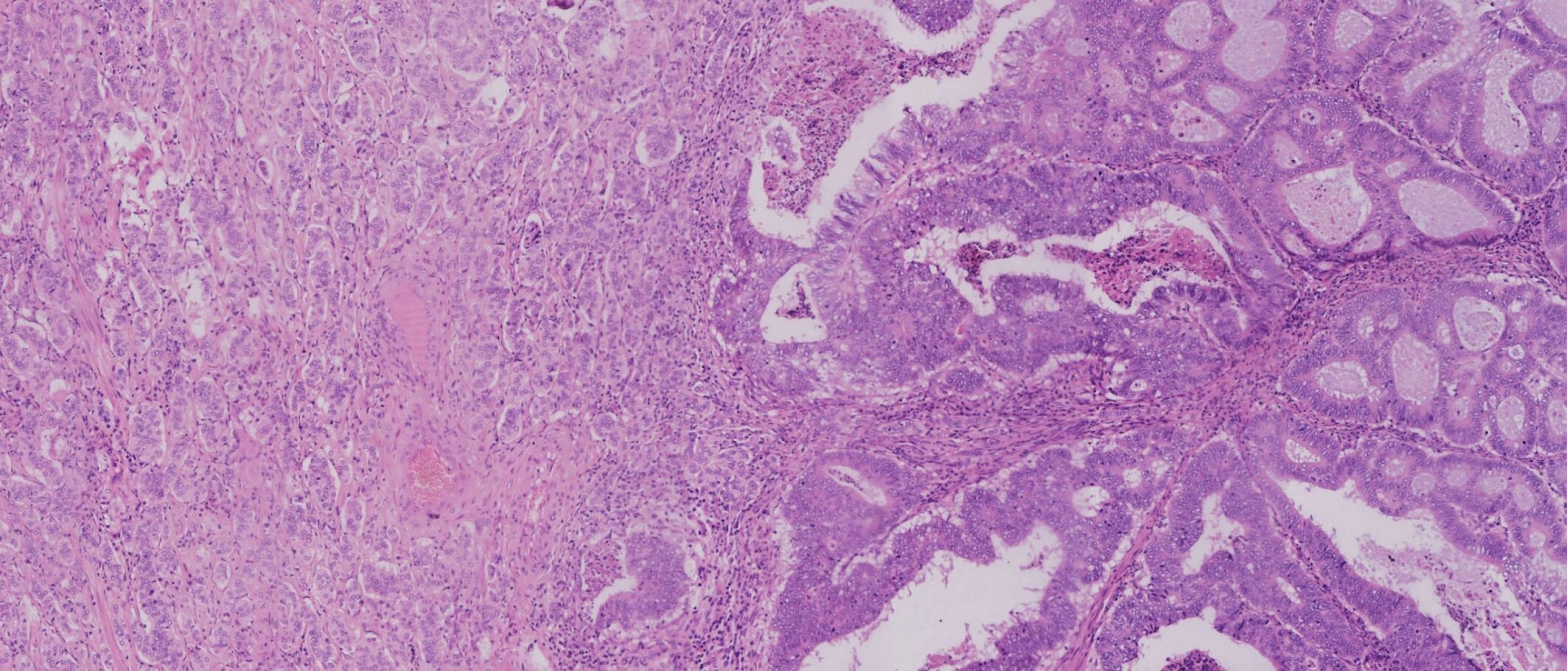
Adenocarcinoma and neuroendocrine tumor (NET) were noted in separate areas, but some areas showed cross-growth.

**Figure 3:**
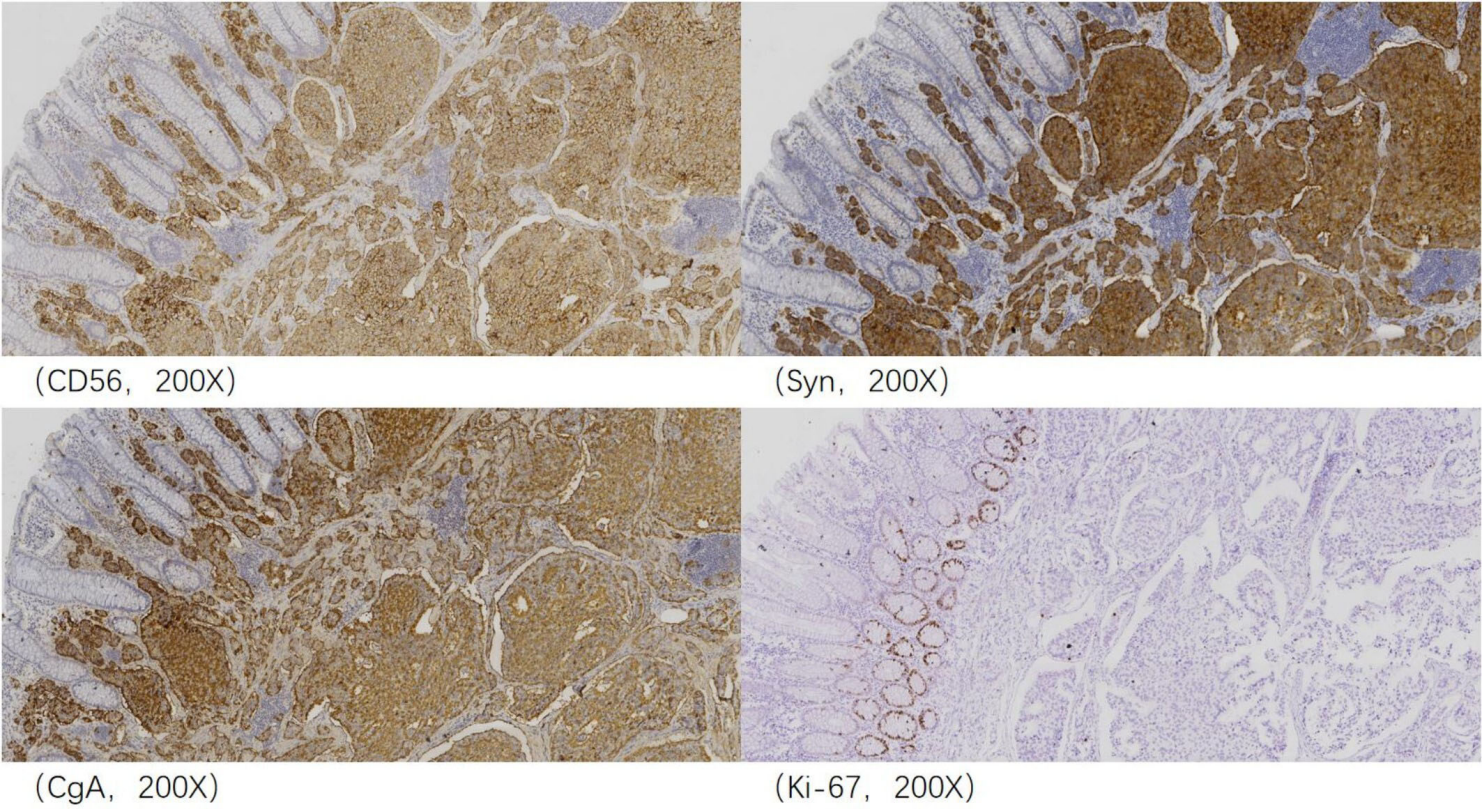
Immunohistochemical analysis revealed that the neuroendocrine component was positive for CD56, Syn, and CgA, and Ki-67 proliferation index was <2% and thus considered neuroendocrine tumor (NET) G1.

**Figure 4:**
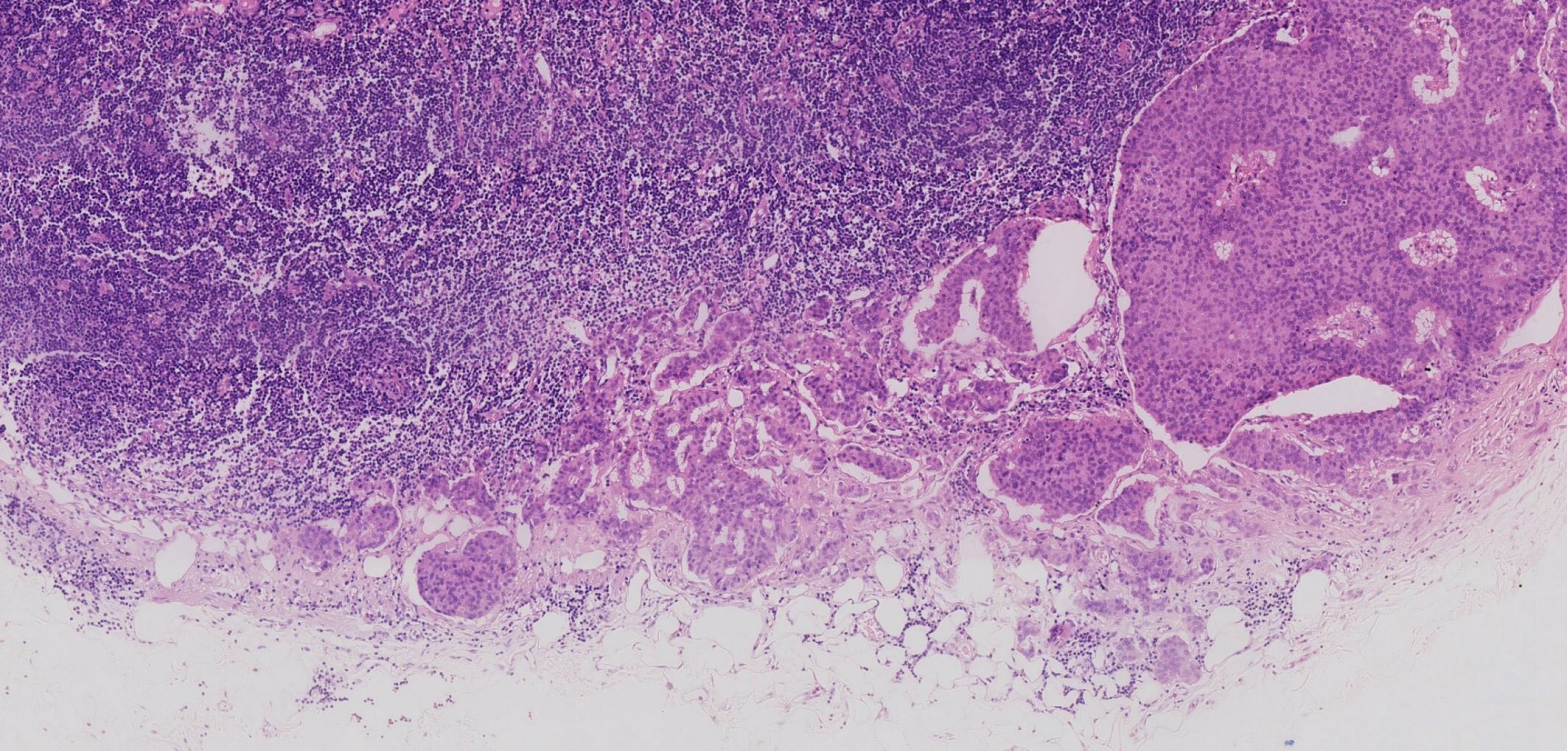
Some lymph nodes around the resected intestinal segment were invaded by neuroendocrine tumor (NET) G1.

The patient underwent total mesorectal excision (TME) and prophylactic ileostomy. Macroscopically, the ulcerative tumor was 4.0 * 3.0 * 1.0 cm in size. The cycle rate was 50% (30 mm/60 mm). The cut surface of the mass was gray-white, solid, and hard and was suspected to invade the deep muscularis propria. Histologically, the tumor was composed of a mixture of two components: moderately differentiated tubular adenocarcinoma (80%) and NET G1 (20%). The two coexisting, distinct tumor types were noted in separate areas, but other areas showed cross-growth (**Figure 2**). Adenocarcinoma invaded the muscularis propria, and the NET component invaded the submucosa in this mixed tumor. The immunohistochemical staining showed that the adenocarcinoma component was positive for CK, P53, and Ki-67. Desmin was positive in muscle tissue. Her-2 was 1+. The cell was also strongly positive for MSH2, MSH6, PMS2, and MLH1, suggesting mismatch repair proficient (pMMR). The other component was slightly positive for CK and Rb and was positive for CD56, Syn, and CgA (**Figure 3**), indicating that it was a neuroendocrine component. Ki-67 proliferation index was <2% (**Figure 3**), so it was diagnosed as NET G1. CD31, CD34, and D2-40 disclosed that NET G1 cells invaded blood vessels and lymphatic vessels, while adenocarcinoma components did not. We performed hematoxylin and eosin (H&E) and Verhoeff’s Van Gieson (EVG) staining to evaluate blood vessels. No abnormality was found. A raised lesion with a diameter of 0.7 cm was seen 2 cm away from the ulcerative tumor, and a nodule with a diameter of 0.5 cm was seen around the intestine. Pathological results confirmed that the raised lesion was a NET G1, invading the submucosa. There were 31 lymph nodes around the resected intestinal segment, of which 11 were invaded by NET G1 (**Figure 4**). No tumor was found in the biopsy of the proximal anastomosis, while a NET G1 was found in the distal anastomosis. In addition, considering that molecular detection of KRAS, NRAS, PIK3CA, and BRAF polygene mutations can predict the drug resistance of patients more accurately and guide clinical medication and scientific selection of treatment plan correctly, we performed KNBP gene mutation detection by amplification refractory mutation system (ARMS) fluorescence quantitative polymerase chain reaction, and no mutations in KRAS, NRAS, PIK3CA, and BRAF genes were detected. These pathological findings supported the diagnosis of adenocarcinoma with NET G1 (pT2N2bM0). The postoperative course was good. Since it was early-stage cancer and ^99m^Tc-octreotide SPECT/CT showed no abnormalities, postoperative adjuvant therapy was not performed.

Five months later, the chest, abdominal, and pelvic enhanced CT showed no metastasis. Multiple red mucosal eminences were found around the anastomosis under the colonoscope. Biopsy was taken, and immunohistochemical staining showed the specimen was positive for Rb, CK, CD56, Syn, CgA, CgB, and SSTR2 and was negative for P53 and S-100. Ki-67 proliferation index was <2%. Therefore, it was consistent with NET G1. Considering that metastasis of NET G1 was found in the lymph nodes around the mixed tumor, we preferred to diagnose these NETs as metastatic tumors rather than independent tumors. Subsequently, the patient underwent ileostomy closure surgery and laparoscopic exploration. At the same time, transanal endoscopic microsurgery (TEM) was performed to remove the lesions found under colonoscopy before the operation. Combined with immunohistochemical staining, the postoperative pathological results showed that they were consistent with NET G1. The patient was followed up every 1–3 months after discharge and did not complain of related discomfort. Chest, abdominal, and pelvic enhanced CT, biochemical examination, blood routine examination, tumor markers, and colonoscopy showed no recurrence and metastasis 6 months after the second operation. Thirteen months after the operation, he is still alive and well.

## 3 Discussion

Due to their rarity, the diagnostic criteria, classification, mechanism, clinical behavior, and treatment options of tumors consisting of two types of components have been controversial. According to the definition, the neuroendocrine component and non-neuroendocrine component must account for more than 30% of the total tumor volume to be diagnosed as mixed neuroendocrine non-neuroendocrine neoplasms (MiNENs) (4). However, the 30% cutoff has been chosen arbitrarily and based on the assumption that a minor component generally does not affect the behavior (5). More and more clinical studies have shown that the 30% threshold may be too high. Previous studies have shown that the neuroendocrine and non-neuroendocrine components may both progress and metastasize independently (6). From this point of view, the natural behavior of this type of tumor is the sum of neuroendocrine and non-neuroendocrine components, not the mean (7). In our case, the proportion of neuroendocrine components in the mixed tumor was only 20%, which did not satisfy the criteria for MiNENs. We just diagnosed the mixed tumor as a sigmoid adenocarcinoma with a focal (20%) NET G1 component. However, it behaved aggressively with metastatic lymph nodes with NET G1 component, which affected the choice of treatment and prognosis to some extent. Fortunately, none of the lymph node metastases presented adenocarcinoma components.

When glandular and endocrine components are present in one tumor, their respective histological patterns are classified as composite tumors, collision tumors, and amphicrines (5). In a composite tumor, the distinct neuroendocrine and non-neuroendocrine components are intermingled. Collision applies when the two components are closely juxtaposed but never intermixed. When dual differentiation was present within the same cell, it can be defined as amphicrine. While the exact pathogenesis of these neoplasms is still a topic of open debate, the tumorigenesis of different types may be distinct (8–10). The first hypothesis about the mechanism of tumorigenesis is that the epithelial and endocrine components descend from different precursor cells independently and grow into the same space by chance. Another hypothesis is that the two components arise from a common multipotent progenitor stem cell with bidirectional differentiation. The third theory proposes that they occur contiguously. The microenvironment is altered by the first neoplasm, leading to the development of an adjacent second neoplasm, which is the result of the progressive accumulation of genetic alterations and aberrations. Finally, an amphicrine tumor is thought to be composed of a single cell type, in which each cell displays both neuroendocrine and non-neuroendocrine phenotypes (9, 10). In our case, the diagnosis of the sigmoid tumor was consistent with a composite tumor since the boundary of the two components was not clear.

Non-neuroendocrine components primarily originate from the mucosa, while neuroendocrine components usually occur in a deeper layer of the colon wall and are easily missed (11). As a result, misdiagnosis and underdiagnosis of the neuroendocrine component are likely to occur. Immunohistochemical study and gene detection play an important role in diagnosis. They are very useful in distinguishing a collision tumor from a composite tumor and in predicting which component contains the more aggressive histological profile. Most available molecular data have been obtained from colorectal mixed adenoneuroendocrine carcinoma (MANEC), in which the adenocarcinoma and NEC components share common driver genetic aberrations. The same results were also found in tumors consisting of adenocarcinomas and NETs (12). This also demonstrates that they originate from a common precursor cell, which undergoes dual differentiation after the first tumorigenic step (7, 13). However, the same conclusion was not confirmed in mixed adenoneuroendocrine tumors (MANETs), a combination of well-differentiated NETs and adenoma. Due to their inertia, those genetic mutations were not detected. Amplifications of KRAS, BRAF V600E, APC, MMR, and HER-2 occur in both components of MiNENs. One series of 44 cases found MMR deficiency in 38.6% of MiNENs. This conclusion increases the possibility of a potential response to programmed death 1 blockade (14). In addition, the molecular mechanisms involved in the occurrence and development of colorectal mixed tumors include some known changes related to the pathogenesis of colorectal adenocarcinoma (10). All these make the targeted therapies possible. In our patients, immunohistochemical study and gene detection played an important role in diagnosis and treatment. The sigmoid tumor was early-stage cancer, and ^99m^Tc-octreotide SPECT/CT showed no abnormalities, so postoperative adjuvant therapy was not performed.

Tumors containing two types of histological components are often diagnosed with extensive lymph node and liver metastasis, which is the most important risk factor for poor prognosis. However, the mechanism driving the transfer remains unclear. A previous retrospective study (6) found that both regional lymph nodes and distant metastases were predominantly invaded by one component. However, distant metastases were mainly invaded by the neuroendocrine component. As the proportion of the neuroendocrine component in the primary tumor increased, the ratio of positive lymph nodes with pure neuroendocrine invasion also increased (6). Sakamoto et al. (15) reported the finding of extramural tumor deposits without lymph node structure (EX) in a patient with rectal NET G1, suggesting that NETs can spread to form EX in a manner similar to colorectal carcinoma, even if it is well differentiated NET G1. Such conclusions indicate that the neuroendocrine component of these special tumors is most likely to be the primary cause of malignancy and should be considered when deciding the appropriate treatment. According to a recent large study (16), there is a significant survival difference among rectal neuroendocrine neoplasm patients with zero positive lymph node, one to four positive lymph nodes, and ≥5 positive lymph nodes. This conclusion should be taken into account when deciding the appropriate treatment of tumors containing neuroendocrine components. Lymph node metastasis of rectal NEN is mainly associated with the number of lesions, the differentiation degree, and the lymphovascular invasion. Multiple lesions can increase the risk of metastasis. A well-differentiated NET, especially the NET G1, rarely metastasizes. In our case, multiple lesions increased the metastasis risk to some extent. Moreover, combined with the hypothesis of the mechanism of a composite tumor, we speculate that the occurrence of lymph node metastasis of NET G1 may be related to the change in the surrounding environment caused by the coexisting adenocarcinoma component. More data are needed to confirm this possibility.

According to the report, in 169 patients with MiNENs or tumors with focal (non-)neuroendocrine component (<30%), only 16% were diagnosed by biopsy; the rest was confirmed through postoperative pathology (6). Thus, it is difficult to diagnose only with biopsy, and this also limits the choice of optimal treatment modalities. Treatment options for tumors containing two types of components are often complex due to their rarity, morphologic diversity, different primary origins, and a low diagnostic rate of preoperative biopsy. Individualized treatment strategies are required. If the neuroendocrine component is poorly differentiated, the tumor is usually treated according to the treatment standard corresponding to pure NEC. Alternatively, when the non-neuroendocrine component is the most represented one and presents more aggressively, the standard of care for epithelial tumors should be applied (8). MANETs have an excellent prognosis, thus not requiring large surgical resection (17). Laenkholm et al. (18) suggested that regardless of the tumor composition, patients should be evaluated for surgical treatment, as it is related to the best prognosis. Patients with disseminated mixed tumors have a very poor prognosis and usually need adjuvant chemotherapy. However, only a few studies on the effect of surgery exist. All of the studies are observational and hold a risk of selection bias, which may overestimate the beneficial effect of surgery (19). In our case, the sigmoid tumor was composed of adenocarcinoma and NET G1, which is a rare mix. Considering that the lesion in the sigmoid was mainly adenocarcinoma with lymph node invasion and the patient was also complicated with colorectal M-NETs, we performed intestinal segment resection on him. Although the main component of the tumor was adenocarcinoma, the component of lymph node metastasis was NET G1, so the prognosis may be better than that of adenocarcinoma metastasis. The patient is still alive and well 13 months after surgery, and further prognosis requires continuous follow-up. A good prognosis also depends on a timely operation. Nevertheless, radical surgery is currently the only hope for a cure and prolonged survival. Regular and adequate follow-up is essential to help rule out metastasis and assess prognosis.

## 4 Conclusion

Tumors composed of two histological components have distinct biological behavior. It is important to elucidate their mechanisms of tumorigenesis by immunohistochemical and gene detection investigations. Rectal M-NETs are associated with a high risk of lymph node metastasis, and treatment should be more radical. In mixed tumors, even well-differentiated NET G1 may metastasize, which may be due to the non-neuroendocrine component that alters the peritumoral environment. Radical surgery is the only hope for a cure currently, and individualized treatment strategies are required.

## Data availability statement

The original contributions presented in the study are included in the article/supplementary material. Further inquiries can be directed to the corresponding authors.

## Ethics statement

The studies involving human participants were reviewed and approved by the ethics committees of Beijing Friendship Hospital, Capital Medical University. The patients/participants provided their written informed consent to participate in this study. Written informed consent was obtained from the individual(s) for the publication of any potentially identifiable images or data included in this article.

## Author contributions

SP and JGS researched the data and wrote the manuscript. KZ and JW reevaluated the pathological results and provided figures for this article. HYZ, YJW, and PL contributed to the discussion. YZ and YDW guided the writing ideas and reviewed the manuscript. All authors agree to be accountable for the content of the work. All authors contributed to the article and approved the submitted version.

## References

[1] Luo C Cen S Ding G Wu W . Mucinous colorectal adenocarcinoma: Clinical pathology and treatment options. Cancer Commun (London England) (2019) 39(1):13. doi: 10.1186/s40880-019-0361-0 PMC6440160 30922401

[2] Nagtegaal ID Odze RD Klimstra D Paradis V Rugge M Schirmacher P . The 2019 who classification of tumours of the digestive system. Histopathology (2020) 76(2):182–8. doi: 10.1111/his.13975 PMC7003895 31433515

[3] Park SS Han N Lee J Chang HJ Oh JH Sohn DK . Multiple small, rectal neuroendocrine tumors with numerous micronests. J Dig Dis (2018) 19(9):572–5. doi: 10.1111/1751-2980.12645 29989305

[4] Washington MK Goldberg RM Chang GJ Limburg P Lam AK Salto-Tellez M . Diagnosis of digestive system tumours. Int J Cancer (2021) 148(5):1040–50. doi: 10.1002/ijc.33210 32674220

[5] Lewin K . Carcinoid tumors and the mixed (Composite) glandular-endocrine cell carcinomas. Am J Surg Pathol (1987) (11 Suppl 1):71–86. doi: 10.1097/00000478-198700111-00007 3544888

[6] Zhang P Li Z Li J Li J Zhang X Lu Z . Clinicopathological features and lymph node and distant metastasis patterns in patients with gastroenteropancreatic mixed neuroendocrine-Non-Neuroendocrine neoplasm. Cancer Med (2021) 10(14):4855–63. doi: 10.1002/cam4.4031 PMC8290235 34109756

[7] Uccella S La Rosa S . Looking into digestive mixed neuroendocrine - nonneuroendocrine neoplasms: Subtypes, prognosis, and predictive factors. Histopathology (2020) 77(5):700–17. doi: 10.1111/his.14178 32538468

[8] Guerrera LP Suarato G Napolitano R Perrone A Caputo V Ventriglia A . Mixed neuroendocrine non-neuroendocrine neoplasms of the gastrointestinal tract: A case series. Healthcare (Basel Switzerland) (2022) 10(4):708. doi: 10.3390/healthcare10040708 35455885 PMC9028985

[9] Kanthan R Tharmaradinam S Asif T Ahmed S Kanthan SC . Mixed epithelial endocrine neoplasms of the colon and rectum - an evolution over time: A systematic review. World J Gastroenterol (2020) 26(34):5181–206. doi: 10.3748/wjg.v26.i34.5181 PMC7495040 32982118

[10] Toor D Loree JM Gao ZH Wang G Zhou C . Mixed neuroendocrine-Non-Neuroendocrine neoplasms of the digestive system: A mini-review. World J Gastroenterol (2022) 28(19):2076–87. doi: 10.3748/wjg.v28.i19.2076 PMC9134131 35664032

[11] de Mestier L Cros J Neuzillet C Hentic O Egal A Muller N . Digestive system mixed neuroendocrine-Non-Neuroendocrine neoplasms. Neuroendocrinology (2017) 105(4):412–25. doi: 10.1159/000475527 28803232

[12] Kasajima A Konukiewitz B Schlitter A Weichert W Klöppel G . NET G3: Clinicopathological and diagnostic features in a consultation case series. Modern Pathology 2020; 33(2):577. doi: 10.1038/s41379-020-0469-4

[13] Sun L Zhang J Wang C Zhao S Shao B Guo Y . Chromosomal and molecular pathway alterations in the neuroendocrine carcinoma and adenocarcinoma components of gastric mixed neuroendocrine-nonneuroendocrine neoplasm. Modern Pathol (2020) 33(12):2602–13. doi: 10.1038/s41379-020-0579-z 32461621

[14] Lou L Lv F Wu X Li Y Zhang X . Clinical implications of mismatch repair deficiency screening in patients with mixed neuroendocrine non-neuroendocrine neoplasms (Minen). Eur J Surg Oncol (2021) 47(2):323–30. doi: 10.1016/j.ejso.2020.08.022 32907775

[15] Sakamoto A Nozawa H Sonoda H Hinata M Ishii H Emoto S . Rectal neuroendocrine tumor with extracapsular lymph node metastasis: A case report. Clin J Gastroenterol (2021) 14(5):1426–30. doi: 10.1007/s12328-021-01447-x 34028785

[16] Fields AC McCarty JC Ma-Pak L Lu P Irani J Goldberg JE . New lymph node staging for rectal neuroendocrine tumors. J Surg Oncol (2019) 119(1):156–62. doi: 10.1002/jso.25307 30481376

[17] La Rosa S Uccella S Molinari F Savio A Mete O Vanoli A . Mixed adenoma well-differentiated neuroendocrine tumor (Manet) of the digestive system: An indolent subtype of mixed neuroendocrine-nonneuroendocrine neoplasm (Minen). Am J Surg Pathol (2018) 42(11):1503–12. doi: 10.1097/pas.0000000000001123 30001239

[18] Laenkholm IT Langer SW Andreassen M Holmager P Kjaer A Klose M . A short report of 50 patients with gastroenteropancreatic mixed neuroendocrine-Non-Neuroendocrine neoplasms (Minen). Acta Oncol (Stockholm Sweden) (2021) 60(6):808–12. doi: 10.1080/0284186x.2021.1903077 33779475

[19] Holmager P Langer SW Kjaer A Ringholm L Garbyal RS Pommergaard HC . Surgery in patients with gastro-Entero-Pancreatic neuroendocrine carcinomas, neuroendocrine tumors G3 and high grade mixed neuroendocrine-Non-Neuroendocrine neoplasms. Curr Treat options Oncol (2022) 23(6):806–17. doi: 10.1007/s11864-022-00969-x 35362798

